# Toward a more reliable characterization of fractal properties of the cerebral cortex of healthy subjects during the lifespan

**DOI:** 10.1038/s41598-020-73961-w

**Published:** 2020-10-12

**Authors:** Chiara Marzi, Marco Giannelli, Carlo Tessa, Mario Mascalchi, Stefano Diciotti

**Affiliations:** 1 Department of Electrical, Electronic, and Information Engineering “Guglielmo Marconi”, University of Bologna, Viale del Risorgimento 2, 40136 Bologna, Italy; 2 Unit of Medical Physics, Pisa University Hospital “Azienda Ospedaliero-Universitaria Pisana”, Pisa, Italy; 3 Division of Radiology, Versilia Hospital, Azienda USL Toscana Nord Ovest, Lido di Camaiore (Lu), Italy; 4 “Mario Serio” Department of Experimental and Clinical Biomedical Sciences, University of Florence, Florence, Italy

**Keywords:** Magnetic resonance imaging, Machine learning, Brain, Predictive markers, Computational science, Biomedical engineering

## Abstract

The cerebral cortex manifests an inherent structural complexity of folding. The fractal geometry describes the complexity of structures which show self-similarity in a proper interval of spatial scales. In this study, we aimed at evaluating in-vivo the effect of different criteria for selecting the interval of spatial scales in the estimation of the fractal dimension (FD) of the cerebral cortex in T_1_-weighted magnetic resonance imaging (MRI). We compared four different strategies, including two a priori selections of the interval of spatial scales, an automated selection of the spatial scales within which the cerebral cortex manifests the highest statistical self-similarity, and an improved approach, based on the search of the interval of spatial scales which presents the highest rounded R^2^_adj_ coefficient and, in case of equal rounded R^2^_adj_ coefficient, preferring the widest interval in the log–log plot. We employed two public and international datasets of in-vivo MRI scans for a total of 159 healthy subjects (age range 6–85 years). The improved approach showed strong associations of FD with age and yielded the most accurate machine learning models for individual age prediction in both datasets. Our results indicate that the selection of the interval of spatial scales of the cerebral cortex is thus critical in the estimation of FD.

## Introduction

The human brain has a complex shape that cannot be comprehensively described in terms of the Euclidean geometry^1^, which is based on simple primitives, such as points, lines, planes, cubes, etc^2^. A complementary and powerful mathematical framework, namely the *fractal geometry*^3^*,* can be exploited in order to properly capture and quantify this level of complexity.

While a fractal object has not a hard-and-fast formal definition^4^, it can be imagined as a rough, fragmented geometric shape whose Euclidean metrics, such as perimeter, area and volume, change depending on the size of the measuring element, rather than tending to a single 'true' value^5^. A fractal object has a property of *self-similarity*, i.e., it can be subdivided into parts, each of which (at least approximately) is a reduced-size copy of the whole^6^. Actually, a strict self-similarity exists only in objects defined by mathematical formulas. On the contrary, real world, natural or computer simulated fractals are self-similar only in a statistical sense and over a limited interval of spatial scales^1^, i.e., they show the *same* statistical distribution of space filling over *some* scales of observation (i.e., not from an infinitesimal to the object size).

In the last 30 years, the fractal analysis has been applied to many structures in the brain^7–9^, including white matter (WM) tracts observed in diffusion-MRI^10^ and cerebral microcirculation^11, 12^. In particular, using T_1_-weighted MRI, fractal properties have been demonstrated in grey matter (GM) and WM surfaces and segmentations (see, e.g.,^5, 13–17^). In this regard, Hofman was one of the first researchers who has shown that the cerebral cortex manifests fractality^16^, and this property was confirmed by other studies, including the works by Free et al*.* and Kiselev et al*.* who demonstrated, using different approaches, that the cerebral cortex presents statistical self-similarity^5, 13^. More recently, the fractal analysis has also been effectively applied to time series of brain signals, e.g., in functional MRI^18–20^.

The most popular index of fractal geometry is the *fractal dimension* (FD), which represents a quantitative measurement of shape complexity^21^. The FD is usually a fractional value^22^ and is imagined as a *dimension* because it provides a measure of space filling-capacity^22^, i.e., it quantifies how much an object fills the space^22, 23^. Indeed, in the same way, we consider the topological dimensions (integer values), which are exponents on length [e.g., a surface area is proportional to length^2^ (topological dimension = 2) or a volume is proportional to length^3^ (topological dimension = 3)]^1^. For example, an FD value within 2 and 3 is typical of a complex and highly folded 2-D surface, like the human cerebral cortex, that is embedded in a 3-D space.

The FD is an extremely compact measure of shape complexity, condensing cortical thickness, sulcal depth, and folding area into a single numeric value—all these indexes being, in fact, closely linked^24^. More importantly, several studies found that information conveyed by FD is additional to that provided by other conventional structural features^5, 24–30^ and that FD is able to detect changes in structural complexity of the cerebral cortex associated with healthy aging^27, 28, 31^ and with neurological diseases^25, 26, 29, 30, 32–37^.

The *box counting algorithm* is the most popular and straightforward implementation used to compute the FD of real objects which present fractal properties in a statistical sense, but are not described by mathematical formulas^24, 26^. At varying the size *s* of an exploring box, this algorithm quantifies the space occupied by the object (i.e., the number of boxes *N(s)* needed to fully cover the object). For a fractal object, the relationship between *log(N(s))* and *log(s)* is linear over an interval of spatial scales. Actually, since the human brain is a real (not mathematical) structure, it exhibits this statistical property only over a limited spatial range, that is defined by a lower (mfs, minimum fractal scale) and an upper (Mfs, maximum fractal scale) limit^38^.

In some previous works^27, 28, 39^, the interval of spatial scales was selected a priori. For instance, Goñi et al.^38^ calculated the FD of the pial surface, i.e., the GM/cerebrospinal fluid (CSF) boundary, of the segmented cerebral cortex and WM, extracted from T_1_-weighted MR images. They chose an a priori range of the box sizes within 5–40% of the smallest Euclidean dimension of the individual’s pial surface^39^. In another recent study, Madan et al. observed that the FD of the cerebral cortex is a marker of structural complexity changes during aging^27^, but the a priori chosen interval of fractal scales was not sufficiently justified^27^.

In other works, the scale range analysis has not been completely selected a priori. In particular, Kiselev et al., analyzing the cerebral cortex of 6 healthy subjects, set a priori the upper limit of the spatial scales equal to the brain size and their results suggested that the cerebral cortex is fractal down to 3 mm, corresponding reasonably well to the cortical thickness^13^. In other studies on the quantification of neuronal dendritic arborizations, the linear region in the log–log plot was identified by computing the n-points local slopes, i.e., the difference in *log(N(s))* divided by *log(s)* for each n successive points. By this way, it was considered the interval of scales in which the local slopes were constant^40, 41^.

In a recent paper, we proposed an automated selection of spatial scales for the computation of the FD of GM and WM using the 3-D box counting algorithm^29^. This method is based on the search of the interval of spatial scales in which the linear regression shows the best fit, as measured by the highest coefficient of determination, adjusted for the number of data points (R^2^_adj_)^29^. Using the FD with automated selection of spatial scales, we showed that the structural complexity of the cerebellum and cerebral cortex was reduced in patients with an inherited form of neurodegenerative disease, namely spinocerebellar ataxia type 2, as compared to a group of healthy controls^29^. Using the same approach, we predicted the cognitive performance in patients with small vessel disease and mild cognitive impairment with different sets of neuroimaging features among which the FD of the cerebral WM was the most relevant^30^. Finally, Krohn et al*.* demonstrated that the same spatial scale optimization can improve accuracy in the FD estimation of the brain in a small sample of healthy controls^31^. In the same work, it has also been shown that the automatically selected scale ranges had a preference for smaller minimum fractal scales and shorter intervals (i.e., number of data points in the log–log plot)^31^.

Given that a minimum span in decades of the interval of scales is needed for an object to be considered fractal (see, e.g.^41^), the assessment of the width of the interval of the automatically selected spatial scales is crucial in establishing FD. So far, this aspect has not sufficiently been investigated for GM and WM structures. In particular, previous literature on automated selection of spatial scales^29–31^ did not explicitly take into account the possibility that the choice of the highest R^2^_adj_ coefficient may be also affected by non-effective decimal places. Given that wider intervals in the log–log plot are preferable to justify the existence of fractal properties, we propose that the automated selection of the spatial scales should be performed by searching for the interval of spatial scales in which the linear regression shows the best fit, as measured by the rounded R^2^_adj_ coefficient, and, in case of equal rounded R^2^_adj_ coefficients, preferring the widest interval.

Conceivably, the employment of different strategies for the selection of the interval of the fractal spatial scales may impact on the FD estimation. Therefore, in this study, we aimed at evaluating in-vivo this effect in the FD estimation of the cerebral cortex in T_1_-weighted MRI. We compared four different strategies, including two a priori selections of the interval of spatial scales, an automated selection of the spatial scales within which the cerebral cortex manifests the highest statistical self-similarity^29, 30^ and an improved approach based on the search of the interval of spatial scales which presents the highest rounded R^2^_adj_ coefficient and, in case of equal rounded R^2^_adj_ coefficient, preferring the widest interval in the log–log plot. This last strategy has been implemented in a Python module, called *fractalbrain,* which is freely available at https://github.com/chiaramarzi/fractalbrain-toolkit (see “Methods” section).

Notably, we employed two public and international datasets of MRI scans for a total of 159 healthy subjects (age range 6–85 years). We computed the association between the different FD estimates and age through the Pearson coefficient of correlation. Thus, we predicted the individual subject age through a machine learning approach (using the out-of-sample error) based on a linear regression in a cross-validation (CV) loop. In particular, we fitted separate regression models for each FD estimate strategy in order to identify the best predictive model (i.e., the strategy for selection of the interval of fractal spatial scales which gives the most accurate predictive age estimates) and compared it with models trained with mean cortical thickness and gyrification index (GI) throughout the cerebral cortex.

## Results

We utilized the scans collected by public and international datasets of 1 mm-isotropic T_1_-weigthed images of healthy children and adolescents [*Nathan Kline Institute (NKI)—Rockland Sample Pediatric Multimodal Imaging Test–Retest Sample*—NKI2 dataset] and adults [*International Consortium for Brain Mapping* (ICBM) dataset] (see “Methods” section). We used the conventional FreeSurfer processing pipeline to perform the cerebral cortical reconstruction of each T_1_-weigthed image and to compute the mean cortical thickness and GI throughout the cerebral cortex (more details in “Methods” section and Supplementary Appendix). Then, we estimated the FD of the cerebral cortex of each subject using our implementation of the 3-D box counting algorithm^29, 30^ and four different strategies for the selection of spatial scales. They include the use of (1) a priori selection of the interval equal to [4 mm–256 mm] (inspired by Kiselev et al.^13^) (Strategy *A priori #1*) (2) a priori selection equal to 5–40% of the smallest Euclidean dimension of the cerebral cortex^39^ (rounded to the nearest power of 2) (Strategy *A priori #2*), (3) an automated selection of spatial scales, within which the cerebral cortex manifests the highest statistical self-similarity^29, 30^ (Strategy *Automated*_*Marzi et al., 2018*_), (4) an improved automated selection of the interval of spatial scales, based on the search of the interval of spatial scales which presents the highest rounded R^2^_adj_ coefficient and, in case of equal rounded *R*^*2*^_*adj*_ coefficient, preferring the widest interval in the log–log plot (Strategy *Automated*_*fractalbrain*_).

**Figure 1:**
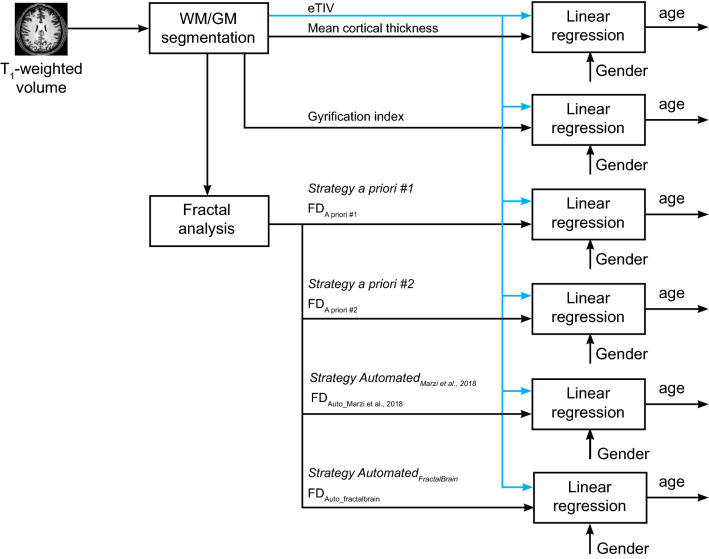
Overview of the feature extraction procedure for individual age prediction through linear regression carried out, separately, for NKI2 and ICBM datasets. We fitted separate regression models for mean cortical thickness and gyrification index throughout the whole cortex and for each FD estimate strategy in order to select the most accurate predictive model. In each model, estimated total intracranial volume (eTIV) values and gender have been inserted as additional features (see the text for other abbreviations).

For both datasets, the quality of the FD estimations has been assessed by individual age prediction through a machine learning approach based on a linear regression in a 5-fold CV loop. We fitted a separate regression model for mean cortical thickness and GI throughout the whole cortex and for each FD estimate strategy, in order to choose the most accurate predictive model. In each model, estimated total intracranial volume (eTIV) values and gender have been inserted as additional features (Fig. 1). The performance has been measured through the mean absolute error (MAE). Since the results may vary depending on how the data are split in each fold of the CV, for each model, we repeated the CV procedure 1000 times. The average MAE from all repetitions was computed to get a final model assessment score. For each model, statistical significance of prediction performance was determined via permutation analysis (further details in “Methods” section).

### NKI2 dataset

The different strategies for FD estimation have been applied on MRI scans of 73 healthy pediatric subjects of the NKI2 dataset, with age ranging from 6 to 17 years (43 males and 30 females, age 11.8 ± 3.1 years, mean ± standard deviation)^42, 43^ (see “Methods” section).

The descriptive statistics of the FD values, mfs, Mfs and interval width obtained for each strategy and the association between FD estimations and age are reported in Table 1.

**Table 1:** Descriptive statistics of the estimated FD values (mean ± standard deviation) in the NKI2 and ICBM datasets using four different strategies for selection of the interval of spatial scales (see the text for details).

Strategy for selection of spatial scales	FD estimates (–)	mfs (mm)	Mfs (mm)	Interval width (decades)	r
**NKI2 dataset**					
A priori #1	2.26 ± 0.02	4 (0) [4, 4]	256 (0) [256, 256]	1.5 (0) [1.5, 1.5]	− 0.15 (0.20)
A priori #2	2.47 ± 0.07	4 (0) [4, 8]	32 (0) [32, 64]	0.9 (0) [0.9, 0.9]	− 0.19 (0.10)
Automated_Marzi et al., 2018_	2.55 ± 0.03	1 (0) [1, 1]	8 (0) [8, 8]	0.9 (0) [0.9, 0.9]	− 0.83 (< 10^–19^)
Automated_fractalbrain_	2.52 ± 0.02	1 (0) [1, 1]	32 (0) [32, 32]	1.5 (0) [1.5, 1.5]	− 0.79 (< 10^–16^)
**ICBM dataset**					
A priori #1	2.26 ± 0.02	4 (0) [4, 4]	256 (0) [256, 256]	1.5 (0) [1.5, 1.5]	− 0.11 (0.30)
A priori #2	2.46 ± 0.06	4 (0) [4, 8]	32 (0) [32, 64]	0.9 (0) [0.9, 0.9]	− 0.09 (0.43)
Automated_Marzi et al., 2018_	2.50 ± 0.02	1 (0) [1, 1]	8 (8) [8, 32]	0.9 (0.3) [0.9, 1.5]	− 0.63 (< 10^–9^)
Automated_fractalbrain_	2.48 ± 0.02	1 (0) [1, 1]	32 (0) [32, 32]	1.5 (0) [1.5, 1.5]	− 0.70 (< 10^–13^)

Minimum (mfs) and maximum (Mfs) fractal scales, as well as the interval width of spatial scales (median (interquartile range) [minimum, maximum]) are also detailed. Finally, the Pearson coefficients of correlation *r* (p-value) between FD estimates and age are shown.

FD: fractal dimension; ICBM: International Consortium for Brain Mapping; mfs: minimum fractal scale; MFS: maximum fractal scale; NKI2: *Nathan Kline Institute—Rockland Sample Pediatric Multimodal Imaging Test–Retest Sample.*

**Figure 2:**
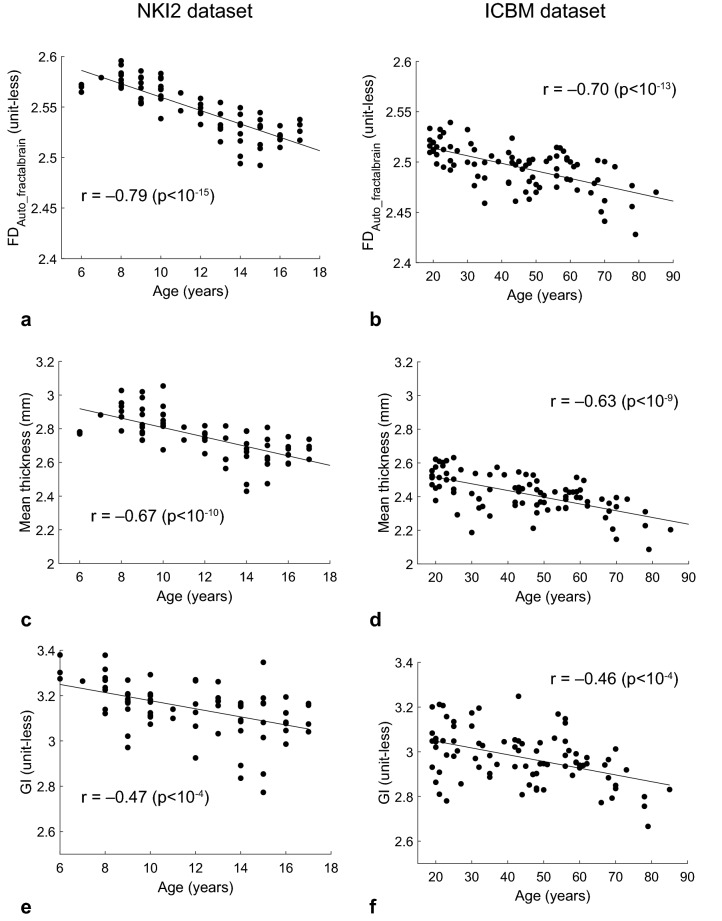
The scatter plots of FD (estimated by using the Strategy *Automated*_*fractalbrain*_) vs. age, mean cortical thickness value vs. age, gyrification index vs. age, in the NKI2 (**a**,**c**,**e**, respectively) and ICBM (**b**,**d**,**f**, respectively) datasets are shown. In each pane, the regression line, the Pearson coefficient of correlation r and the relative p-value are also reported (see the text for abbreviations).

The intervals width of spatial scales obtained with the improved approach are larger than those obtained with the Strategy *Automated*_*Marzi et al., 2018*_. Indeed, the intervals selected with the Strategy *Automated*_*fractalbrain*_ covered 1.5 (0) decades [median(interquartile range)], while the Strategy *Automated*_*Marzi et al., 2018*_ selected intervals covering 0.9 (0). The Pearson coefficient of correlation between FD values and age was significant (p < 10^–15^) only for the two strategies with automated selection of spatial scales. The significant correlation coefficients were markedly negative (*r* = − 0.83 and *r* = − 0.79) indicating that FD (i.e., complexity) reduces during brain development. For the Strategy *Automated*_*fractalbrain*_, the scatter plot of FD values vs. age along with the regression line are shown in Fig. 2a. As a reference, the scatter plots of mean cortical thickness vs. age and GI vs. age are reported in Fig. 2c,e (Pearson correlation coefficient *r* = − 0.67 and *r* = − 0.47, respectively). The scatter plot of FD_A priori#1_ vs. age and FD_A priori#2_ vs. age are shown in Supplementary Figure S1 (panes a and c).

The quality of the FD estimations has been assessed by individual age prediction through a machine learning approach. Among all models, the MAE value was lowest for the Strategy *Automated*_*fractalbrain*_ (MAE = 1.29 years). All MAE values are provided in Table 2.

**Table 2:** Individual age prediction in NKI2 and ICBM datasets using different sets of input features: the average MAE values (using 1000 repetitions of the 5-fold CV) between predicted values of test set of the 5-fold CV and actual values are provided.

Features^a^	Average MAE^b^ (years)
**NKI2 dataset**	
CT	1.75
GI	1.81
FD_A priori#1_	2.04
FD_A priori#2_	2.37
FD_Auto_Marzi et al., 2018_	1.43
FD_Auto_fractalbrain_	1.29
**ICBM dataset**	
CT	12.0
GI	14.2
FD_A priori#1_	15.8
FD_A priori#2_	15.8
FD_Auto_Marzi et al., 2018_	12.3
FD_Auto_fractalbrain_	11.1

CT: mean cortical thickness; ICBM: International Consortium for Brain Mapping; FD_A priori#1_: fractal dimension computed using the a priori method #1 for selection of spatial scales; FD_A priori#2_: fractal dimension computed using the a priori method #2 for selection of spatial scales; FD_Auto_fractalbrain_: fractal dimension computed using the automated selection of the spatial scales as in Strategy *Automated*_*fractalbrain*_; FD_Auto_Marzi et al., 2018_: fractal dimension computed using the automated selection of the spatial scales as in Strategy *Automated*_*Marzi et al., 2018*_; GI: gyrification index; MAE: mean absolute error; NKI2: *Nathan Kline Institute—Rockland Sample Pediatric Multimodal Imaging Test–Retest Sample.*

^a^In all models, estimated intracranial volume (eTIV) values and gender were also included as input features.

^b^All the models have a p-value < 0.0002. They have been computed using 5000 permuted-data CV scores simulating the null distribution.

### ICBM dataset

The different strategies for FD estimation have also been applied on the *International Consortium for Brain Mapping* (ICBM) dataset. It is composed of MRI examinations of 86 healthy adult and elderly subjects with age ranging from 19 to 85 years (41 males and 45 females, age 44.2 ± 17.1 years, mean ± standard deviation) (see “Methods” section).

The descriptive statistics of the FD values, mfs, Mfs and interval width obtained for each strategy and the association between FD estimations and age are reported in Table 1. The intervals width of spatial scales obtained with the improved approach are larger than those obtained with the Strategy *Automated*_*Marzi et al., 2018*_. Indeed, the intervals selected with the Strategy *Automated*_*fractalbrain*_ covered 1.5 (0) decades [median (interquartile range)], while the Strategy *Automated*_*Marzi et al., 2018*_ selected intervals covering 0.9 (0.3). The Pearson coefficient of correlation between age and FD values was significant (p < 10^–9^) only for the two strategies with automated selection of spatial scales. These significant correlation coefficients were markedly negative (*r* = − 0.63 and *r* = − 0.70) indicating that FD (i.e., complexity) reduces during healthy aging. Among these values, the Strategy *Automated*_*fractalbrain*_ showed the highest correlation coefficient in absolute value, namely − 0.70. For this strategy, the scatter plot of FD values vs. age along with the regression line are shown in Fig. 2b. As a reference, the scatter plots of mean cortical thickness vs. age and GI vs. age are reported in Fig. 2d,f (Pearson correlation coefficient *r* = − 0.63 and *r* = − 0.46, respectively). The scatter plot of FD_A priori#1_ vs. age and FD_A priori#2_ vs. age are shown in Supplementary Figure S1 (panes b and d).

As for the NKI2 dataset, the quality of the FD estimations has been assessed by individual age prediction through a machine learning approach (Fig. 1). Among all FD estimates, the MAE value was the lowest for the Strategy *Automated*_*fractalbrain*_(MAE = 11.1 years). All MAE values are provided in Table 2.

## Discussion

The fractal geometry is able to capture the inherent structural complexity of the cerebral cortex which has proven to show statistical self-similarity^3^. The FD is a unit-less index which measures the space-filling capacity of complex and statistical self-similar objects, i.e., of structures which have the same statistical distribution of space filling at varying the scale of observation, providing a quantitative index of the structural complexity^44, 45^. In this study, we applied the box counting algorithm for estimating FD^29^ and compared four different strategies for selection of the interval of spatial scales. These strategies include two a priori selections, inspired by previous literature^13, 39^, an automated selection of the spatial scales within which the cerebral cortex manifests the highest statistical self-similarity^29, 30^ and an improved approach, based on the search of the interval of spatial scales which presents the highest rounded R^2^_adj_ coefficient and, in case of equal rounded R^2^_adj_ coefficient, preferring the widest interval in the log–log plot. In particular, the improved approach has been proposed taking into account that a wider width of the interval of the spatial scales is crucial to justify the existence of fractal properties^46^ and the choice of this interval should not be guided by non-effective *R*^*2*^_*adj*_ decimal places. We employed two public and international datasets of in-vivo MRI scans for a total of 159 healthy subjects for the study of brain development and aging. Notably, different strategies for the selection of the interval of the fractal scales produced different FD estimates. Moreover, when an automatic strategy was applied, the determined optimal range of spatial scales can vary across subjects. From a methodological point of view, the approach of Strategy *Automated*_*fractalbrain*_ has the potential to improve the reliability of FD estimation. Accordingly, we found that this improved approach consistently showed strong association of FD estimates with age, that exceeded those of mean cortical thickness and GI. We have also shown that the prediction error obtained using the FD values with Strategy *Automated*_*fractalbrain*_ was the lowest among those obtained using all other FD estimates, mean cortical thickness or also GI, in both datasets.

The significant negative correlations we found between FD values of the cerebral cortex and age in the NKI2 and ICBM datasets suggest an overall reduction of the structural complexity starting in childhood and continuing in adulthood and aging.

Indeed, postmortem studies have found that aging is associated to loss of neuropil and nerve fibers, with a reduction of dendrites, spines density and synapses, together with a direct loss of neurons (the latter relatively limited in healthy aging)^47–49^, and all these changes could reduce complexity and potentially modify brain fractal properties. In this regard, we performed a comparison with the gyrification index – a common index of structural complexity of the human cortex. Indeed, althought FD and GI measure the structural complexity of the cortex from different viewpoints^5^, our results suggest that they share a common decreasing trend with age. In fact, in both datasets, we found a steady decrease of the GI with age, in agreement with previous literature. Indeed, the GI increases in the very first few years of life^50, 51^. Then, starting from the age of 4 years, the GI steadily decreases with age^52–55^. In a review by Zilles et al.^56^, the decrease of the GI with age since childhood has been hypothesized to be due to pruning with programmed cell death and reduction of cell numbers and connectivity^57^ and to the increasing gyral width driven by the peak of myelination^58, 59^. In principle, these anatomical changes could also underly a decrease in structural complexity detectable by FD analysis.

So far, a few studies evaluated the possible changes with age of the FD of the normal brain in children and adolescents^60^, adults^61^ or elderly subjects^27^.

Blanton et al*.* evaluated the FD of the pial surface of four regions (inferior and superior frontal lobe, temporal lobe and parieto-occipital lobe) in children and adolescents of 6–16 years of age and reported a significant increase in cortical complexity with age in frontal lobe regions only^60^. They supposed that the increasing structural complexity of the frontal lobe during the development and adolescence may be referred to the emergence of the executive functions, as planning and abstract reasoning, that typically emerge at these ages^60^. For the sake of a direct comparison, we applied our newly proposed approach (Strategy *Automated*_*fractalbrain*_) to the same regions considered by Blanton et al. (see Supplementary Appendix). This novel analysis confirmed an overall decreasing trend of FD with age in all areas (see Supplementary Figure S2). Differences between our results and those obtained by Blanton et al.^60^ may be due to several reasons. Firstly, they used two different imaging acquisition protocols which could have introduced some bias. Moreover, these protocols were sub-optimal, because voxel size was anisotropic and slice thickness (1.2 and 1.4 mm) appreciably greater than the common 1 mm value used in 3D T_1_-weighted sequences. Secondly, they traced cortical sulci for delimiting boundaries of various lobes completely in a manual manner. Thirdly, from a methodological point of view, they calculated the FD of the pial surface, while the superiority of the FD of the entire volume of the cerebral cortex as compared to the FD of the pial surface has been previously shown^25, 26^. Fourtly, Blanton et al.^60^, used the algorithm for FD estimation adopted in^62^, which, unfortunately, lacks many information, e.g., if they used the box-counting, the range of spatial scales, the minimum number of data points, etc. Finally, we wish to point out that Blanton et al.^60^ analyzed a small (n = 24) sample of normal children and adolescents.

Madan et al.^27^, utilizing a sample of 427 individuals (20–80 years old) from the freely available IXI (“Information eXtraction from Images”) dataset found that the FD of the cerebral cortex was more sensitive to age-related differences than other metrics of cortical morphology such as cortical thickness or gyrification. This could reflect the fact that the FD of the cerebral cortex is related to both cortical thickness and folding properties of the cortex, summarizing in a single quantitative index the structural complexity characteristics of the cerebral cortex^24^. Madan et al.^27^ estimated the FD using the dilation method, a procedure alternative to the box-counting algorithm, and using a subsample of the entire IXI dataset composed of 581 adults. In particular, they removed 6 subjects for whom the gyrification index analyses failed to determine a suitable convex-hull surface for at least one hemisphere and 130 subjects for whom the surface reconstruction failed at visual inspection, and apparently did not use of any form of surface correction. In our study, we carefully corrected the surface errors following the recommendations of the FreeSurfer developers, removing only four subjects of the NKI2 dataset in our analysis (see “Methods” section for details). Thus, we included also those subjects showing a cerebral cortex more challenging for the segmentation process, an occurrence that could be age- and complexity-related.

Concerning the a priori strategies for the selection of interval of fractal spatial scales used in this study, we referred to the hypothesis and findings from Kiselev et al.^13^ and Goni et al.^39^, but we did not apply the methods for FD estimation they proposed.

The used interval of spatial scales in fractal analysis is crucial in brain applications, as shown in this study. Indeed, while the interval of spatial scales is not limited for a truly mathematical fractal structure, its actual assessment is a fundamental prerequisite for a reliable and proper estimation of FD^38^ of the cerebral cortex, as well as of other biological structures. The interval of spatial scales determines the fractal nature of a structure, based on the Mandelbrot's assertion that "*fractals are not a panacea; they are not everywhere*"^63^. The minimum width of the interval of spatial scales for which an object may be considered fractal has been widely debated. In biological structures, the range of spatial scales is often rather limited, mainly comprised between 0.5 and 2 decades, as compared to other structures like coastlines or clouds^46, 64, 65^. Moreover, a spatial scale range of more than two orders of magnitude is rarely observed in biological structures^1, 22, 65, 66^. For these reasons, as minimum range of spatial scales, we chose the smallest interval greater than 1 decade^41^, i.e., 1.2 decades, corresponding to 5 data points in the log–log plot.

In this study, the widths of intervals of spatial scale obtained with the proposed improved automatic approach (Strategy *Automated*_*fractalbrain*_) were larger than those obtained with the Strategy Automated_Marzi et al., 2018_, i.e., 1.5 vs. 0.9 decades (median value) in both datasets, thus clearly supporting the existence of fractal properties for the cerebral cortex.

In particular, from a biological viewpoint, the existence of a minimum fractal scale reflects the fact that, theoretically, at increasing the magnification, the effect of increasing detail (as occurs for pure fractals) may vanish, or substantially change, because the morphology of the constituent elements, such as, neurons, becomes evident^64^. However, it is not conceivable that this effect may be relevant for typical voxel size of isotropic high-resolution T_1_-weighted MRI, i.e., 1 mm. Indeed, for all subjects of both datasets, the minimum fractal scale was 1 mm. In this regard, further studies are needed to explore the hypothesis that the cerebral cortex can still present fractal properties for a lower fractal scale and to assess whether the cerebral cortex can show statistical self-similarity for a wider interval of spatial scale than that observed so far. In other words, due to limitations in spatial resolution of conventional MRI acquisitions, we can probe only a few of the spatial scales of observations in which the cerebral cortex might exhibits statistical self-similarity. Further insights into fractal properties of the cerebral cortex might be obtained by using T_1_-weighted MRI data, with higher spatial resolution, acquired on ultra-high field (7 T) scanners.

In conclusion, by using two large datasets of healthy subjects and machine learning models for individual age prediction, we showed that the selection of the interval of spatial scales is important for a more adequate characterization of fractal properties of the cerebral cortex, as well as for a more reliable estimation of FD. Furthermore, this study indicates a monotonic decrease in structural complexity (in terms of FD) of the cerebral cortex with age during almost all the lifespan.

## Methods

### NKI2 dataset

The NKI2 public dataset (https://fcon_1000.projects.nitrc.org/indi/CoRR/html/nki_2.html) is composed of MRI scans of 77 healthy pediatric subjects with age ranging from 6 to 17 years (45 males and 32 females, age 11.7 ± 3.2 years, mean ± standard deviation). It has been focused on creating resource for studying developmental trajectories of brain function and structure and relationships with phenotypic measurements. The NKI2 dataset includes a retest scan session for 70 participants. All subjects underwent high resolution T_1_-weighted imaging (with voxel size = 1.0 mm × 1.0 mm × 1.0 mm), functional resting state, task based functional MRI (fMRI), diffusion weighted imaging and arterial spin labeling perfusion scans on a 3 T scanner. In this study, only the T_1_-weighted MR images from the first scan were used. All the scans were acquired on the same scanner and with the same protocol. More details about scan acquisition parameters can be found in Nooner et al.^42^. The FreeSurfer segmentation procedure (see Cortical reconstruction and segmentation of the cerebral cortex section below), after manual editing up to 3 times (see Supplementary Appendix), failed for four subjects (IDs: A00052165, A00054578, A00055907, A00055908) who were thus excluded from further analysis. For this reason, in this study, we analyzed the remaining 73 subjects (43 males and 30 females, age 11.8 ± 3.1 years, mean ± standard deviation, age ranging from 6 to 17 years).

### ICBM dataset

We used public data collected by the ICBM and belonging to the *1000 Functional Connectomes Project* (freely accessible at https://fcon_1000.projects.nitrc.org/). This project has the purpose of providing to the broader imaging community a complete access to a large-scale functional imaging dataset. The ICBM dataset is composed of MRI examinations of 86 healthy subjects with age ranging from 19 to 85 years (41 males and 45 females, age 44.2 ± 17.1 years, mean ± standard deviation). All subjects underwent high resolution T_1_-weighted imaging (with voxel size = 1.0 mm × 1.0 mm × 1.0 mm) and resting state fMRI on a 3 T scanner. In this study, T_1_-weighted MR images were employed only. All the scans have been acquired on the same scanner and with the same protocol. More details about scan acquisition parameters can be found in Mazziotta et al.^67^. No subject has been excluded after quality assessment of FreeSurfer segmentation outputs (see Cortical reconstruction and segmentation of the cerebral cortex section below).

### Cortical reconstruction and segmentation of the cerebral cortex

For each subject, cortical reconstruction and volumetric segmentation of the cerebral cortex was performed with the FreeSurfer image analysis suite v.5.3^68^ (https://surfer.nmr.mgh.harvard.edu/). Mean cortical thickness and GI throughout the cerebral cortex have also been recorded from FreeSurfer outputs. More details about FreeSurfer analysis and procedures for quality assessment of the cerebral cortical segmentations have been reported in the Supplementary Appendix.

### Computation of the FD of the cerebral cortex with a 3-D box counting algorithm

**Figure 3:**
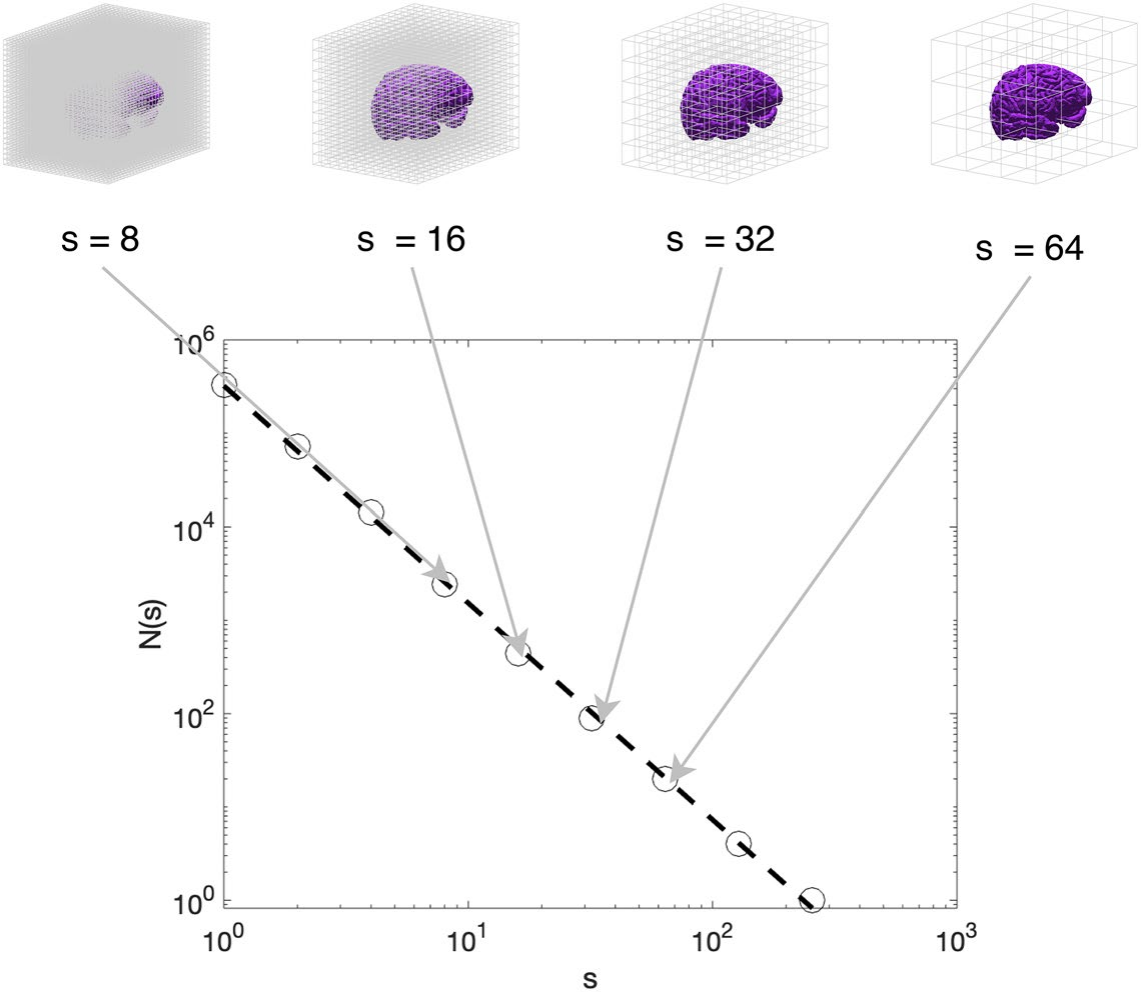
An example of the grid superimposed on a 3-D binary image in the 3-D box counting algorithm: the size *s* of the cubes contained in each grid (e.g., s = 8, 16, 32, 64 voxels) determines the value of the count *N(s)* in the log–log plane. The figure has been prepared with Matlab R2019b (MathWorks, Natick, MA, US).

We computed the FD of the cerebral cortex through the 3-D box counting algorithm^69^, as previously described^29, 30^. Briefly, let *I(x,y,z)* be a 3-D binary segmentation of the cerebral cortex. A grid containing 3-D boxes of side *s* was superimposed on *I(x,y,z)* and the number of boxes *N(s)* needed to fully cover the object was counted. The procedure was repeated for various *s* values (Fig. 3). In order to prevent a systematic influence of the grid placement on the object contained in *I(x,y,z)*^4, 70, 71^, for each *s* value, 20 uniformly distributed random offsets were applied on the grid origin and the relative box counts have been averaged to obtain a single *N(s)* value^39^. For a fractal object, the data points of *N(s)* versus *s* within a certain interval of spatial scales in the log–log plane can be modeled by a linear regression function. Once the minimum and maximum fractal scales interval have been selected, either a priori or automatically (see next section), the FD was computed as the absolute value of the slope of the regression line estimated within the selected spatial scales^3^. An example of the procedure is shown in Fig. 3.

This linear relationship in the log–log plane corresponds, in the natural scale, to a power law *N(s)* = C * s ^*−FD*^, where FD is the exponent (with a negative sign) and *C* is the prefactor^3^. Given that the linear regression is computed in a log–log plot, the box counting algorithm was applied by using a uniform sampling of the spatial scales in the log–log plane, which corresponds to an exponential sampling in the natural scale, i.e., using *s* = *2*^* k*^ mm, with *k* = {0, 1, …,8}.

### Strategies for selection of the interval of spatial scales

We estimated the FD of the cerebral cortex of each subject using four different strategies for the selection of spatial scales on both NKI2 and ICBM datasets. They include the use of an a priori spatial scales range with minimum and maximum fractal scales inspired by Kiselev et al*.* (Strategy *A priori #1*)^13^, and by Goñi et al*.* (Strategy *A priori #2*)^39^ (rounded to the next power of 2). In particular, Kiselev et al. have proposed a range of spatial scales from 3 mm (a typical value of cortical thickness) to 25 cm (the head size), while Goñi et al. have suggested to take into account the values between 5 and 40% of the smallest Euclidean dimension of the object^39^. We estimated the smallest Euclidean dimension of the cortical ribbon segmentation by firstly computing its bounding box, i.e., the box with the smallest volume that fully encloses the cortical ribbon. Then, we derived the 5% and 40% of the shortest side length (rounded to the nearest power of 2) and set them as the minimum and maximum fractal scales, respectively.

As third strategy, we adopted an automated selection of spatial scales within which the cerebral cortex manifests the highest statistical self-similarity^29, 30^ (Strategy *Automated*_*Marzi et al., 2018*_*).* This algorithm has been previously described in detail and validated on synthetic phantoms^30^. Briefly, we selected the interval of spatial scales in which *I(x,y,z)* manifests more marked fractal properties, identifying the range of *s* values in which the linear regression shows the highest coefficient of determination (adjusted for the number of data points) *R*^2^_*adj*_, used as a goodness-of-fit indicator. To this end, we carried out the linear regression for each combination of minimum and maximum spatial scales allowed. We considered a minimum number of 4 data points in the log–log plane in order to explore a sufficient range of spatial scales (in the worst case, i.e., mfs = 1 mm (for k = 0) and Mfs = 8 mm (for k = 3), the interval covers ≈ 0.9 decades) for which a fractal object can be defined. This third strategy does not take into account the possibility that the choice of the highest *R*^*2*^_*adj*_ value may be affected by non-effective decimal places. Given that it is theoretically better to privilege the widest possible spatial interval^41, 72^, we propose a forth strategy based on an improved automated selection of the spatial scales (Strategy *Automated*_*fractalbrain*_). As most statistical measurements, only a few decimal places of *R*^*2*^_*adj*_ are actually effective, contributing to the significant figures^73^. Since it can be interpreted as a proportion of explained variance, i.e., a percentage value, we followed the common recommendations suggesting that percentage values over 90% (in our study, highest *R*^*2*^_*adj*_ were > 0.90 for each subject) may need one decimal place^73^. One decimal place in percentage is equivalent to 3 decimal places of the *R*^*2*^_*adj*_ in natural scale—thus we rounded the *R*^*2*^_*adj*_ using 3 decimal places. Given that wider intervals in the log–log plot are preferable to justify the existence of fractal properties, in the Strategy *Automated*_*fractalbrain*_, the automated selection of the spatial scales is performed by searching for the interval of spatial scales in which the linear regression shows the best fit, as measured by the rounded *R*^*2*^_*adj*_ coefficient and, in case of equal rounded *R*^*2*^_*adj*_ coefficients, by selecting the widest interval (i.e., the one that contains as many points as possible in the log–log plot). In this way, we prevent that scale selection might be guided by non-effective *R*^*2*^_*adj*_ decimal places, and prefer wider intervals in the case of equal (rounded) *R*^*2*^_*adj*_. In order to explore at least 1 decade^41^, we considered a minimum number of 5 data points in the log–log plane—in the worst case the interval covers ≈ 1.2 decades which can occur, e.g., for mfs = 1 mm (for k = 0) and Mfs = 16 mm (for k = 4) or mfs = 2 mm (for k = 1) and Mfs = 32 mm (for k = 5)).

**Figure 4:**
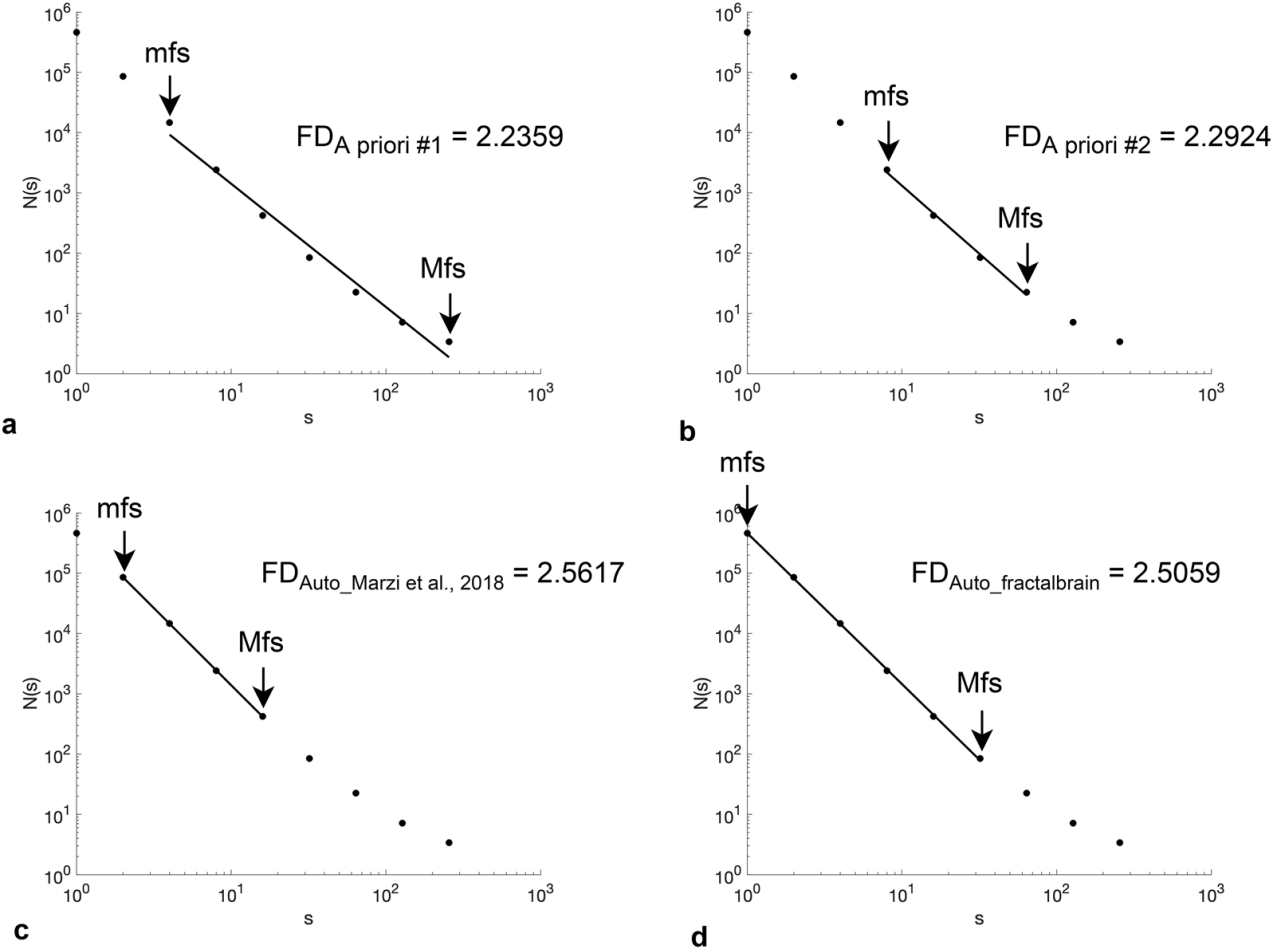
The selection of the minimum and maximum fractal scales and the corresponding FD value have been reported for one subject of ICBM dataset (ID sub94945) for (**a**) strategy *A priori #1*; (**b**) strategy *A priori #2*; (**c**) strategy *Automated*_*Marzi  et al., 2018*_; and (**d**) strategy *Automated*_*fractalbrain*_.

An example of how the four strategies affects the linear regression fit in the log–log plot is shown in Fig. 4.

### Statistical analysis

For each dataset and for each strategy used for selecting the spatial scales interval, we computed the descriptive statistics of FD values of the cerebral cortex along with that of mfs, Mfs and interval width. In order to assess the potential use of the FD as a neuroimaging marker of brain development and aging, we performed a linear regression analysis and computed the Pearson’s correlation coefficient between FD measurements (assessed using the 4 strategies) and age, separately, in both the NKI2 and ICBM datasets. For all tests, a p-value < 0.05 was considered statistically significant. As a reference, the correlation analysis between mean cortical thickness and age and between GI and age have also been carried out.

### Individual age prediction

We fitted a separate regression model for mean cortical thickness and GI throughout the whole cortex and for each FD estimate strategy in order to choose the most accurate model in predicting individual age using the ordinary least-square linear regression. In each model, eTIV values and gender have been inserted as additional features (Fig. 1). For each model, to evaluate the performance on unseen data, the regression task was performed in a 5-fold CV loop. Before each training of the linear regression in CV, each feature was standardized with reference to the training set only. Test set data were not used in any way during the learning process, thus preventing any form of the peeking effect^74^. Performance was quantified in terms of the MAE between predicted and actual age values computed on the test set of the CV. Given that the results may vary depending on how the data are split in each fold of the CV, for each model, we repeated the CV procedure 1000 times. The average MAE from all repetitions was computed to get a final model assessment score. For each model, statistical significance of prediction performance was determined via permutation analysis—recommended especially when the sample size is small^75^. Thus, for each model, 5000 new models were created using a random permutation of the labels (i.e., individual age), such that the input features were dissociated from its corresponding individual age, to simulate the null distribution of the performance measure against which the observed value was tested^76^. MAEs were considered significant if the p-value computed using permutation tests was < 0.05. We used own code developed in Python programming language (release 3.7.1, available at https://www.python.org/) for data analysis.

## Supplementary information

Supplementary Information.

## Data Availability

The datasets analysed during the current study are freely available online: NKI2 dataset: https://fcon_1000.projects.nitrc.org/indi/CoRR/html/nki_2.html. ICBM dataset: ICBM repository, https://fcon_1000.projects.nitrc.org/fcpClassic/FcpTable.html.
