# Supplementary material for: Toward a more reliable characterization of fractal properties of the cerebral cortex of healthy subjects during the lifespan
Source: Sci Rep. 2020 Oct 12;10:16957. doi: 10.1038/s41598-020-73961-w (PMC7550568; doi:10.1038/s41598-020-73961-w)
Supplement: Supplementary file 1 — Supplementary Information. [file 41598_2020_73961_MOESM1_ESM.doc]

**Toward a more reliable characterization of fractal properties of the cerebral cortex of healthy subjects during the lifespan**

Chiara Marzi^1^, Marco Giannelli^2^, Carlo Tessa^3^, Mario Mascalchi^4^, Stefano Diciotti^1*^

^1^ Department of Electrical, Electronic, and Information Engineering “Guglielmo Marconi”, University of Bologna, Bologna, Italy

^2^ Unit of Medical Physics, Pisa University Hospital “Azienda Ospedaliero-Universitaria Pisana”, Pisa, Italy

^3^ Division of Radiology, Versilia Hospital, Azienda USL Toscana Nord Ovest, Lido di Camaiore (Lu), Italy

### ^4^ “Mario Serio” Department of Experimental and Clinical Biomedical Sciences, University of Florence, Florence Italy

* **Corresponding Author:** Prof. Stefano Diciotti, Department of Electrical, Electronic, and Information Engineering “Guglielmo Marconi”, University of Bologna, Viale del Risorgimento 2, 40136, Bologna, Italy. E-mail: stefano.diciotti@unibo.it

*Cortical ribbon segmentation*

Completely automated cortical reconstruction of each subject’s structural T_1_-weighted MRI scan was performed by employing the *FreeSurfer* image analysis suite (http://surfer.nmr.mgh.harvard.edu/)[^1^](#_ENREF_1), a dedicated brain segmentation software[^2-7^](#_ENREF_2). Briefly, this includes removal of non-brain tissue using a hybrid watershed/surface deformation procedure, automated Talairach transformation, segmentation of the subcortical white matter and deep gray matter volumetric structures, intensity normalization, tessellation of the gray/white matter boundary, automated topology correction[^8^](#_ENREF_8) and surface deformation following intensity gradients to optimally place the gray/white and gray/cerebrospinal fluid borders at the location where the greatest shift in intensity defines the transition to the other tissue class. Once the cortical models are complete, a number of deformable procedures can be performed for further data processing and analysis including surface inflation, registration to a spherical atlas which is based on individual cortical folding patterns to match cortical geometry across subjects, parcellation of the cerebral cortex into units with respect to gyral and sulcal structure[^9^](#_ENREF_9), and creation of a variety of surface based data including maps of curvature and sulcal depth. More details of the segmentation steps of FreeSurfer have been described previously and an example of the pial surface reconstruction is shown in Supplementary Fig. S3.

We also exploited the capabilities within the FreeSurfer suite to quantify local cortical gyrification (lGI) following a surface-based approach[^16^](#_ENREF_16). Briefly, in each vertex, a spherical region of interest is delineated on an outer envelope (ROI_O_) that tightly wraps the pial cortical surface, and its corresponding region of interest on the pial cortical surface (ROI_P_) is identified using a matching algorithm based on geodesic constraints. Thus, the lGI is derived as the ratio between ROI_P_ and ROI_O_ areas quantifying the amount of cortex buried within the sulcal folds in the surrounding spherical region. Then, we averaged the lGI within the entire cortex for obtaining a gyrification index (GI) representative of the cortical complexity of each subject.

For the sake of a direct comparison of the FD using our newly proposed approach (Strategy *Automated_fractalbrain_*) to the same areas of Blanton et al.[^17^](#_ENREF_17), in the NKI2 dataset, we also computed the bilateral segmentation of the inferior and superior frontal lobes using the Desikan-Killiany parcellation – in particular, the inferior frontal lobe has been formed by merging the pars opercularis, pars triangularis, and pars orbitalis[^9^](#_ENREF_9). Parietal, temporal and occipital lobar segmentations have been carried out using the “strict” lobar definition adopted in FreeSurfer (https://surfer.nmr.mgh.harvard.edu/fswiki/CorticalParcellation).

Cortical reconstruction and GM/WM surfaces were manually inspected for defects. The correction techniques recommended by *FreeSurfer* developers, including editing of brain/WM masks as well as adding control points and re-running of the *FreeSurfer* pipeline (https://surfer.nmr.mgh.harvard.edu/fswiki/FsTutorial/TroubleshootingData), were applied. All views (coronal, sagittal and axial) were used to confirm segmentation errors. The manual editing and re-running were carried out up to 3 times to assure that all defects were corrected in all datasets.

Finally, for each subject, we converted the cortical ribbon in a binary image, in order to estimate the FD value.


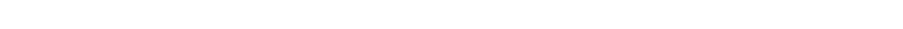

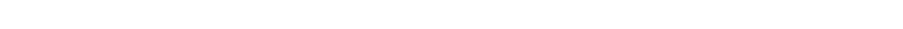


**Supplementary Figure S1.** The scatter plot of FD_A priori#1_ vs. age and FD_A priori#2_ vs. age in the NKI2 (panes a and c, respectively) and in ICBM (panes b and d, respectively) datasets are shown. In each pane, the regression line, the Pearson coefficient of correlation r and the relative p-value are also reported (see the text for abbreviations).

*
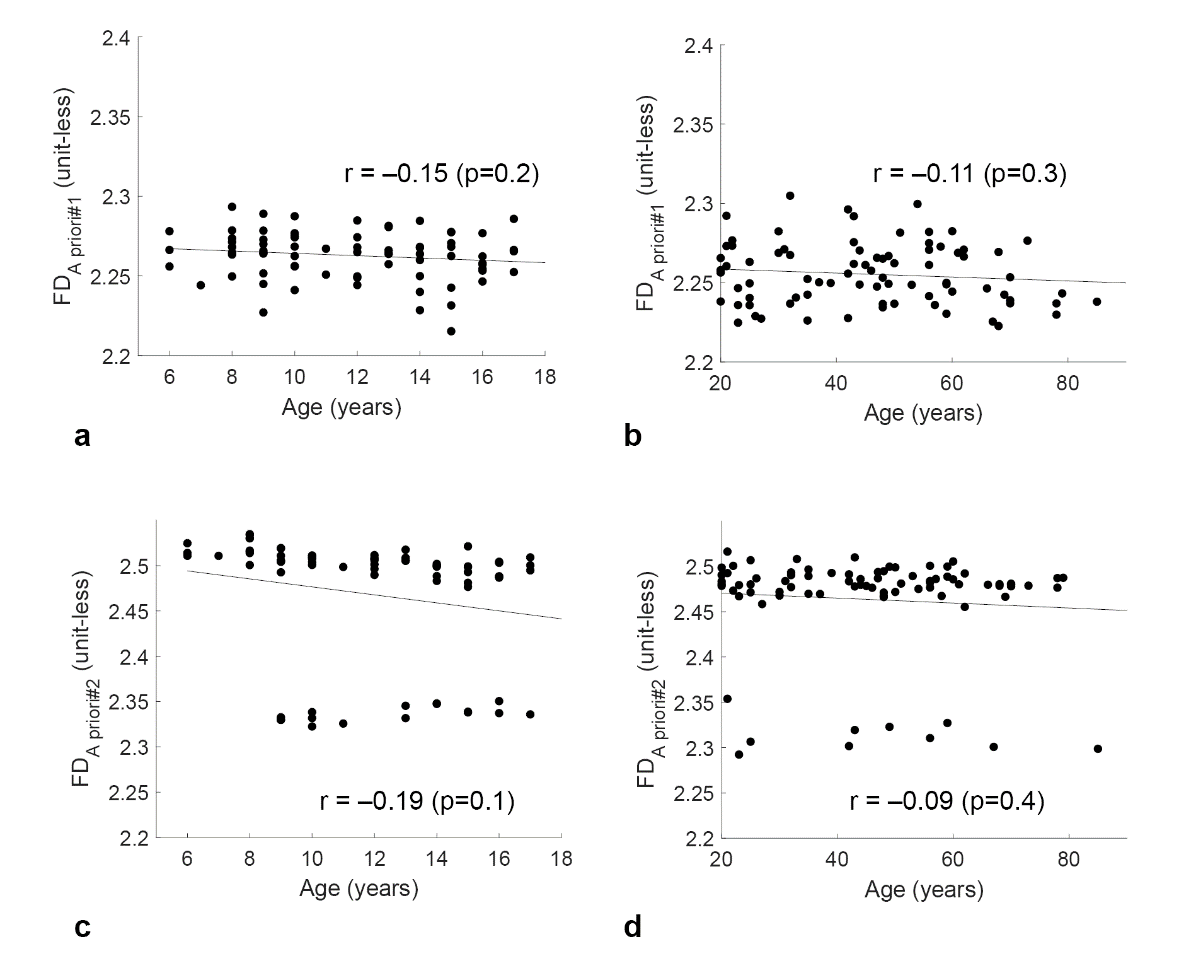
*

**Supplementary Figure S2.** The scatter plots of FD (estimated by using the Strategy *Automated_fractalbrain_*) vs. age in the same area of those computed in Blanton et al.[^17^](#_ENREF_17) are shown for the NKI2 dataset. In particular, the areas include the left and right inferior (a-b) and superior frontal lobe (c-d), the temporal lobe (e-f), the parietal lobe (g-h) and the occipital lobe (i-j).


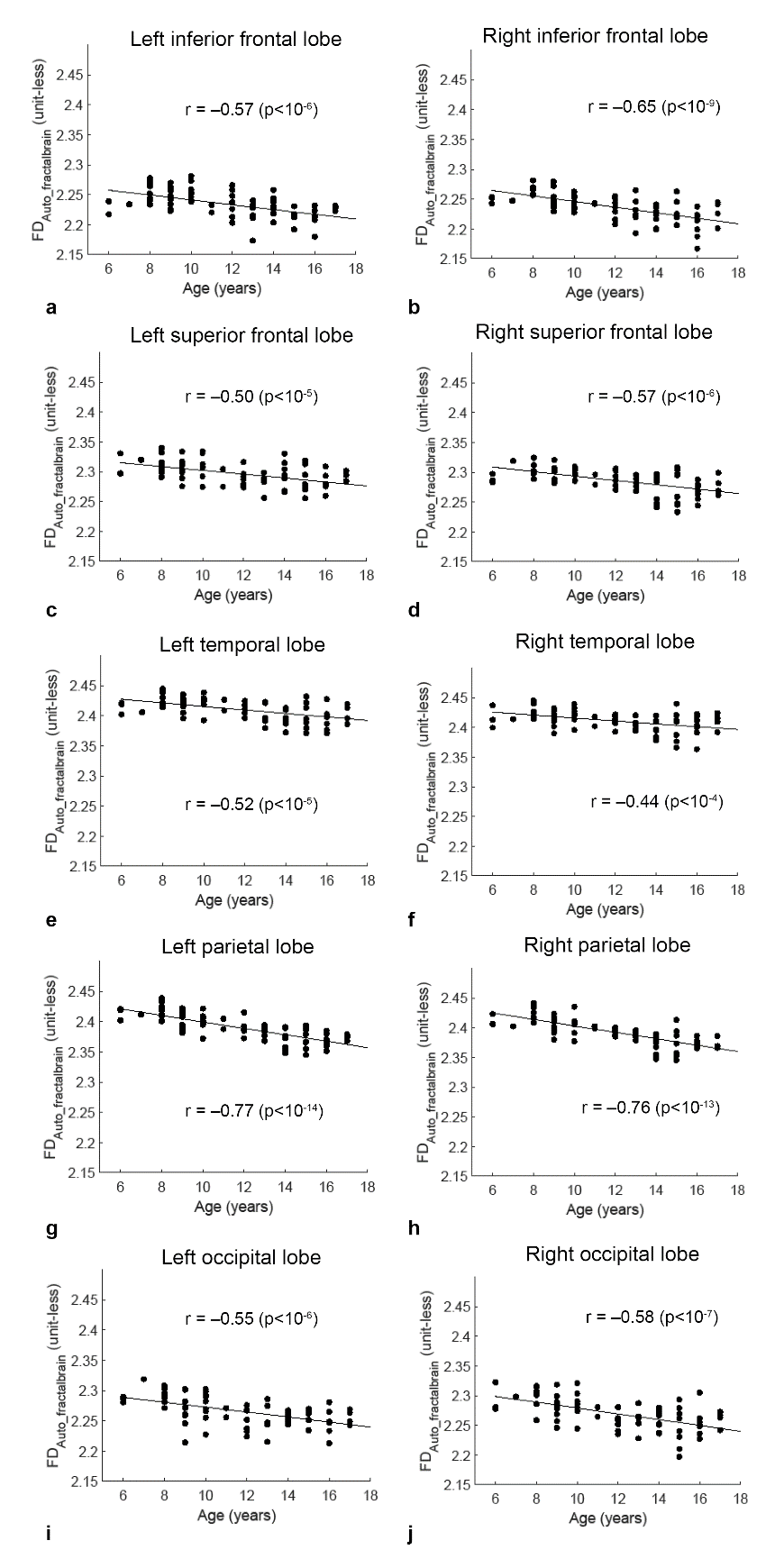


**Supplementary Figure S3.** A 3-D view of the pial surface of one subject from the ICBM dataset (ID sub00448) is shown: gyri and sulci are represented in green and red, respectively. The figure has been prepared with *freeview*, a tool included in the *FreeSurfer* suite.


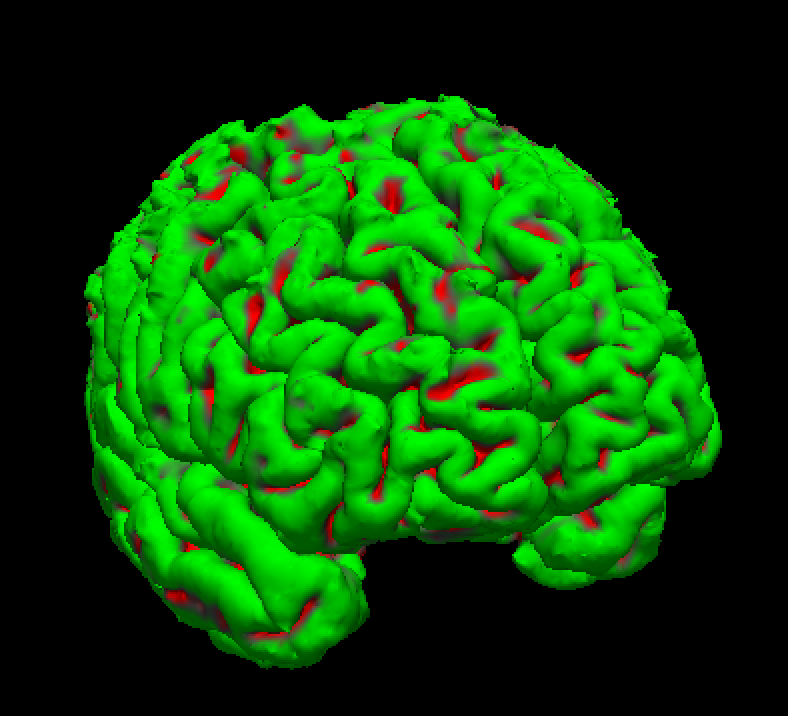


*References*

1 Fischl, B. FreeSurfer. *Neuroimage* **62**, 774-781, doi:10.1016/j.neuroimage.2012.01.021 (2012).

2 Rosas, H. D. *et al.* Regional and progressive thinning of the cortical ribbon in Huntington’s disease. *Neurology* **58**, 695-701 (2002).

3 Han, X. *et al.* Reliability of MRI-derived measurements of human cerebral cortical thickness: The effects of field strength, scanner upgrade and manufacturer. *Neuroimage* **32**, 180-194 (2006).

4 Lee JK., L. J., Kim JS., Kim IY., Evans AC., Kim SI.. A novel quantitative cross-validation of different cortical surface reconstruction algorithms using MRI phantom. *Neuroimage* **31**, 572-584 (2006).

5 Kang, X., Herron, T. J., Cate, A. D., Yund, E. W. & Woods, D. L. Hemispherically-unified surface maps of human cerebral cortex: reliability and hemispheric asymmetries. *PLoS One* **7**, e45582, doi:10.1371/journal.pone.0045582 (2012).

6 Keller, S. S. *et al.* Voxel-based statistical analysis of fractional anisotropy and mean diffusivity in patients with unilateral temporal lobe epilepsy of unknown cause. *J. Neuroimaging* **23**, 352-359, doi:10.1111/j.1552-6569.2011.00673.x (2013).

7 King, R. D. Computation of Local Fractal Dimension Values of the Human Cerebral Cortex. *Applied Mathematics* **5**, 1733-1740 (2014).

8 Fischl, B., Liu, A. & Dale, A. M. Automated manifold surgery: constructing geometrically accurate and topologically correct models of the human cerebral cortex. *IEEE Medical Imaging* **20**, 70-80 (2001).

9 Desikan, R. S. *et al.* An automated labeling system for subdividing the human cerebral cortex on MRI scans into gyral based regions of interest. *Neuroimage* **31**, 968 - 980 (2006).

10 Dale, A., Fischl, B. & Sereno, M. I. Cortical Surface-Based Analysis: I. Segmentation and Surface Reconstruction. *Neuroimage* **9**, 179 - 194 (1999).

11 Fischl, B., Sereno, M. I. & Dale, A. Cortical Surface-Based Analysis: II: Inflation, Flattening, and a Surface-Based Coordinate System. *Neuroimage* **9**, 195 - 207 (1999).

12 Fischl, B. *et al.* Whole brain segmentation: automated labeling of neuroanatomical structures in the human brain. *Neuron* **33**, 341-355 (2002).

13 Fischl, B. *et al.* Sequence-independent segmentation of magnetic resonance images. *Neuroimage* **23**, S69 - S84 (2004).

14 Jovicich, J. *et al.* Reliability in multi-site structural MRI studies: Effects of gradient non-linearity correction on phantom and human data. *Neuroimage* **30**, 436 - 443 (2006).

15 Segonne, F., Pacheco, J. & Fischl, B. Geometrically accurate topology-correction of cortical surfaces using nonseparating loops. *IEEE Trans. Med. Imaging* **26**, 518-529 (2007).

16 Schaer, M. *et al.* A surface-based approach to quantify local cortical gyrification. *IEEE Trans. Med. Imaging* **27**, 161-170, doi:10.1109/TMI.2007.903576 (2008).

17 Blanton Rebecca E. *et al.* Mapping cortical asymmetry and complexity patterns in normal children. *Psychiatry Research Neuroimaging* (2001).
